# Integrative Mapping of SNHG1 RNA–Chromatin Contacts onto the Cancer-Specific Super-Enhancer Landscape in HCT116 Colorectal Cancer Cells

**DOI:** 10.3390/ijms27083642

**Published:** 2026-04-19

**Authors:** Grigory K. Ryabykh, Ekaterina D. Osintseva, German A. Ashniev, Yulia V. Makus, Alexey V. Orlov, Petr I. Nikitin, Natalia N. Orlova

**Affiliations:** 1 Vavilov Institute of General Genetics, Russian Academy of Sciences, 3 Gubkina St., 119991 Moscow, Russia; ryabykhgrigory@gmail.com; 2 Prokhorov General Physics Institute of the Russian Academy of Sciences, 38 Vavilov St., 119991 Moscow, Russia; osintseva.ed@gmail.com (E.D.O.); ashnievh@gmail.com (G.A.A.); ivmakus@edu.hse.ru (Y.V.M.); nikitin@kapella.gpi.ru (P.I.N.); 3 Central University, 7 Gasheka St., 123056 Moscow, Russia

**Keywords:** long non-coding RNA, SNHG1, super-enhancer, RNA–chromatin interactions, histone modifications, epigenetic regulation, colorectal cancer, HCT116

## Abstract

Long non-coding RNAs (lncRNAs) interact with chromatin and recruit epigenetic complexes to specific genomic loci, yet their relationship with super-enhancers (SEs), key regulatory elements frequently reprogrammed in cancer, remains unexplored. We developed an integrative pipeline that combines RNA–chromatin contact data (RNA-Chrom), histone modification–lncRNA expression correlation profiles (HiMoRNA peaks), and super-enhancer annotations (SEdb 3.0) to map lncRNA–SE regulatory axes. Applying this framework to SNHG1 in HCT116 colorectal cancer cells, we identified 21 SNHG1-reactive super-enhancers (Ψ-SEs) among 184 cancer-specific SEs, at which SNHG1 physical contacts co-occur with SNHG1-correlated histone modifications (HiMoRNA peaks), predominantly H3K4me1 (permutation *p* = 0.001, fold enrichment = 2.03). Comparison with 4145 lncRNAs demonstrated that epigenetic correlations alone do not distinguish SNHG1; instead, the addition of the contact layer is required to delineate the Ψ-SE set. Differential expression (DESeq2) and co-expression analyses in 471 TCGA-COAD tumor samples identified 12 Ψ-SE target genes (including CDC20, PDP1, and TOP1) consistently upregulated in both HCT116 cells and patient tumors and positively correlated with SNHG1, with the co-expression signal robust to tumor purity correction. The proposed Ψ/Ω classification provides a generalizable framework for prioritizing super-enhancers at which lncRNA–chromatin interactions may shape the local epigenetic environment across cancer types.

## 1. Introduction

Super-enhancers (SEs) are large clusters of transcriptional enhancers that drive the expression of genes critical for cell identity and fate determination [1,2,3,4]. Distinguished from typical enhancers by their extended size, high density of transcription factor binding, and elevated levels of mediator complex occupancy, SEs exert disproportionately strong effects on transcriptional output [5,6,7,8]. In cancer, SEs are frequently acquired *de novo* or reprogrammed to activate oncogenes, making them central elements of the malignant transcriptional program [3,9,10]. The systematic cataloguing of SEs across cell types in databases such as SEdb 3.0 has enabled genome-wide analyses of SE-associated regulatory landscapes [11].

Epigenetic modifications at SEs—particularly histone marks such as H3K27ac, H3K4me1, and H3K4me3—serve as hallmarks of their activity state and regulatory potential [12,13]. Changes in these modifications at SE loci have been linked to altered expression of downstream target genes in both normal development and disease [3,14,15]. However, the factors that shape the epigenetic landscape around SEs remain incompletely understood, especially regarding the role of non-coding RNA (lncRNAs) species in modulating chromatin states at these regulatory elements [16]. lncRNAs have emerged as important regulators of chromatin architecture and gene expression [16,17]. Through direct interaction with chromatin, lncRNAs can recruit activating or repressive complexes to specific genomic loci, thereby influencing the local epigenetic environment [18]. Recent experimental and computational advances now enable genome-wide mapping of RNA–chromatin interactions and their correlation with histone modification patterns [19,20]. The RNA-Chrom database provides one-to-all RNA–chromatin contact maps for numerous lncRNAs across cell types [21], while the HiMoRNA database quantifies correlations between lncRNA expression levels and histone modification signals at specific genomic loci [22].

Among lncRNAs implicated in cancer, SNHG1 (Small Nucleolar RNA Host Gene 1) is notable for its consistent upregulation across multiple tumor types [23,24] and its predominantly nuclear localization, which is dynamically regulated and functionally significant [25,26]. Located at chromosome 11q12.3, SNHG1 serves as a precursor for eight small nucleolar RNAs (SNORD22, SNORD25-31), yet its mature spliced transcript exhibits independent regulatory functions which suggest a broad capacity for epigenetic modulation. Despite these advances, whether SNHG1 RNA–chromatin contacts preferentially target SE regions and influence their epigenetic state and downstream gene expression has not been systematically examined.

In this study, we integrate SNHG1 RNA–chromatin contact data (RNA-Chrom), histone modification–lncRNA expression correlation profiles (HiMoRNA), and super-enhancer annotations (SEdb 3.0) to map the SNHG1–SE regulatory axis in HCT116 colorectal cancer cells. We show that SNHG1 contacts a defined set of HCT116-specific super-enhancers and that a substantial fraction of these SEs carry histone modification signatures that covary with SNHG1 expression—predominantly H3K4me1, an enhancer-associated mark present at active as well as primed/permissive enhancers. On this basis, we classify the contacted SEs into SNHG1-reactive (Ψ-SE) and SNHG1-independent (Ω-SE) categories. Among 4145 lncRNAs in the HiMoRNA database, epigenetic correlations alone do not distinguish SNHG1, but the addition of the RNA–chromatin contact layer identifies a significantly enriched subset of 21 Ψ-SEs. Target genes of Ψ-SEs validated across both HCT116 and TCGA-COAD include established colorectal cancer-associated genes such as CDC20, PDP1, and TOP1. These findings position SNHG1 as a candidate epigenetic modulator at super-enhancer loci and provide a resource for prioritising lncRNA–SE interactions for experimental validation.

## 2. Results and Discussion

### 2.1. Integrative Mapping of SNHG1 Contacts onto the HCT116 Super-Enhancer Landscape

**Figure 1:**
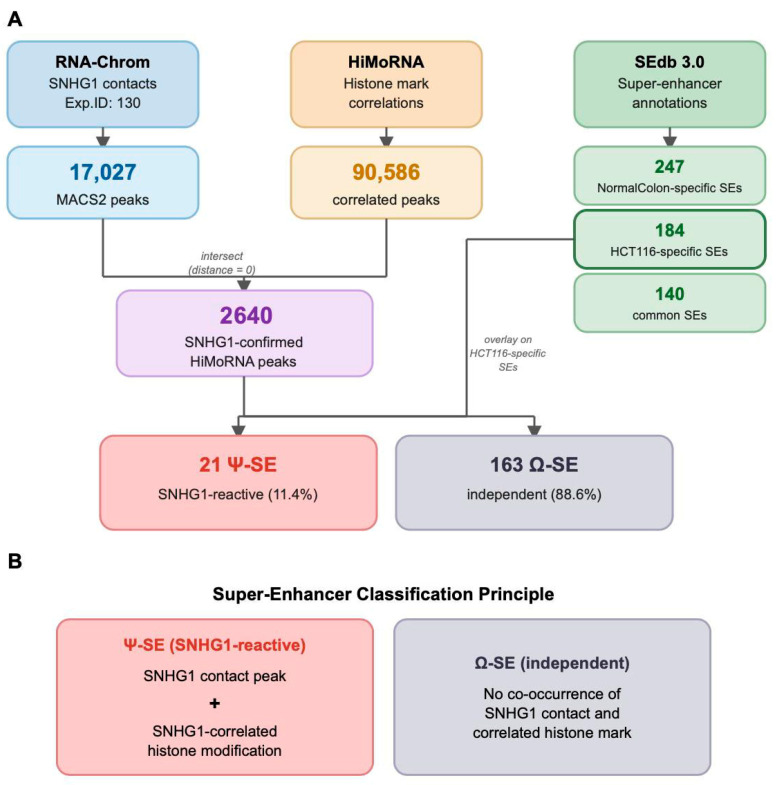
Overview of the integrative analysis. (**A**) Data integration workflow for classification of SNHG1-associated super-enhancers. (**B**) Classification scheme for super-enhancers based on SNHG1-confirmed epigenetic marks.

To investigate whether SNHG1 RNA–chromatin contacts coincide with epigenetically active super-enhancer regions in colorectal cancer cells, we developed an integrative pipeline combining three publicly available data resources (Figure 1A). The analysis proceeded through three stages: characterisation of the SNHG1 contact landscape, construction of a cancer-specific super-enhancer map, and integration of epigenetic correlation data.

MACS2 peak calling on SNHG1 one-to-all RNA–chromatin contact data from the RNA-Chrom database (Exp. ID: 130) yielded 17,027 high-confidence contact peaks distributed across all autosomes and the X chromosome. This broad genomic distribution is consistent with a trans-acting regulatory capacity [16], as previously suggested by SNHG1’s predominantly nuclear localisation (~70%) and its documented interactions with chromatin-modifying complexes. SNHG1 is consistently upregulated across colorectal cancer cohorts and is abundantly expressed in HCT116 cells (mean TPM ≈ 120), consistent with the extensive chromatin contact profile observed.

In parallel, a consensus super-enhancer map was constructed by merging three independent HCT116 SE annotations and six normal colon tissue annotations from SEdb 3.0 (see Section 3). The resulting 571 consensus SEs were classified by tissue specificity: 184 HCT116-specific, 247 normal colon-specific, and 140 shared. The substantial fraction of cancer-specific SEs (32.2%) is consistent with the established role of super-enhancer reprogramming in malignant transformation and defines the SE space within which SNHG1 interactions were assessed.

To link SNHG1 contacts with epigenetic states, we leveraged the HiMoRNA database, which catalogues genomic loci where histone modification levels significantly correlate with lncRNA expression across cell types and tissues. For SNHG1, the database contains 90,586 such peaks (Table S1). We intersected these with the 17,027 SNHG1 contact peaks and found that 2640 HiMoRNA peaks (2.9%) directly overlapped with SNHG1 contacts. These 2640 loci represent sites where SNHG1 is physically present and where histone modification levels covary with SNHG1 expression—a conjunction that would be expected if SNHG1 participates in shaping the local chromatin environment at these sites.

A permutation test confirmed that SNHG1 contacts are significantly enriched at super-enhancer loci. Among 184 HCT116-specific SEs, 79 (42.9%) overlapped with at least one SNHG1 contact peak, compared to an expected 51.1 ± 5.7 SE overlaps per iteration when 17,027 peaks were randomly shuffled across the genome 1000 times (range: 34–68; *p* < 0.001; fold enrichment = 1.55). These data indicate that SNHG1 chromatin contacts are non-randomly distributed with respect to the cancer-specific super-enhancer landscape.

To determine whether this enrichment is specific to super-enhancers or extends to enhancer elements more broadly, we intersected SNHG1 contacts with H3K27ac and H3K4me1 ChIP-seq peaks for HCT116 (ENCODE; ENCSR000EUT, ENCSR000EUS) after excluding SE regions. SNHG1 contacts were significantly enriched at both H3K27ac-defined (fold = 2.02, *p* < 0.001) and H3K4me1-defined (fold = 2.00, *p* < 0.001) typical enhancers (Table S2), comparable to or exceeding the enrichment observed at super-enhancers. This broad enhancer association underscores that the specificity of the Ψ-SE classification derives from the triple intersection rather than from contact enrichment alone.

### 2.2. A Defined Subset of Cancer-Specific Super-Enhancers Carries SNHG1-Associated Epigenetic Signatures

**Figure 2:**
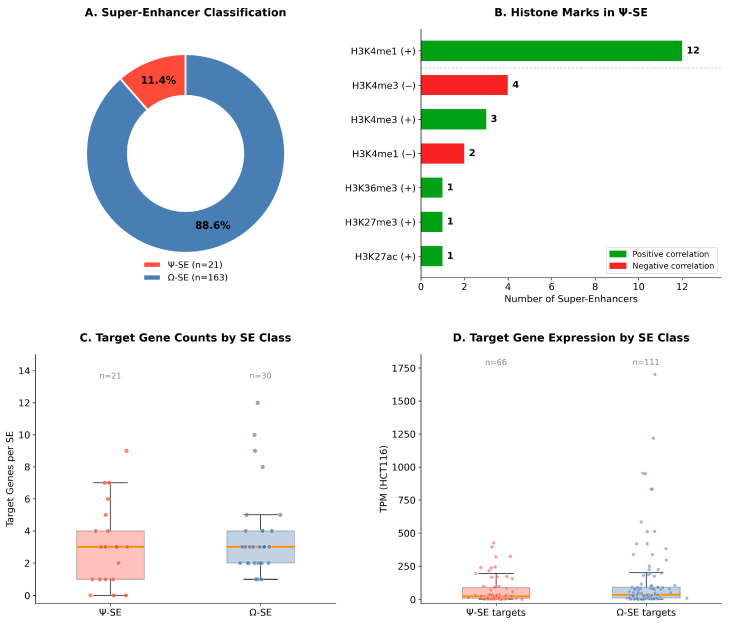
Classification and histone modification landscape of SNHG1-associated super-enhancers. (**A**) Proportion of Ψ-SEs and Ω-SEs among HCT116-specific SEs. (**B**) Distribution of histone modification correlations within Ψ-SEs. (**C**) Number of target genes per SE for Ψ-SEs and Ω-SEs with SNHG1-correlated HiMoRNA marks at target gene TSSs. (**D**) Expression levels (TPM) of target genes associated with Ψ-SEs and the Ω-SE–TSS subset.

Overlaying the 2640 SNHG1-confirmed HiMoRNA peaks onto the 184 HCT116-specific super-enhancers revealed that 21 SEs (11.4%) harboured at least one such peak within their boundaries. We designated these as Ψ-SE (Psi; SNHG1-reactive)—super-enhancers at which SNHG1 physical contact co-occurs with SNHG1-correlated histone modifications. The remaining 163 SEs (88.6%) were designated Ω-SE (Omega; SNHG1-independent) (Figure 1B and Figure 2A; the genomic coordinates are provided in Table S3).

**Figure 3:**
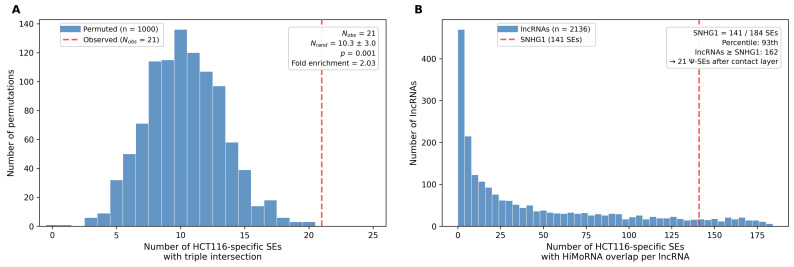
Significance and specificity of the SNHG1–HiMoRNA–SE triple intersection. (**A**) Permutation test: observed vs. expected number of Ψ-SEs. (**B**) HiMoRNA–SE overlap across 2136 lncRNAs; SNHG1 ranks in the 93rd percentile.

A joint permutation test confirmed that this triple intersection is statistically significant. In 1000 iterations in which SNHG1 contact peaks were randomly repositioned across the genome, the mean number of HCT116-specific SEs carrying confirmed HiMoRNA peaks was 10.4 ± 3.0 (range: 1–20), compared to the observed 21 (*p* = 0.001, fold enrichment = 2.03; Figure 3A). The Ψ-SE set thus represents a significantly enriched subset of cancer-associated super-enhancers at which SNHG1 physical contact co-occurs with correlated epigenetic modification.

Importantly, this enrichment depends on the integration of both data layers. To assess whether HiMoRNA correlations alone are sufficient to distinguish SNHG1, we examined HiMoRNA peaks for all 4145 lncRNAs catalogued in the HiMoRNA database and counted, for each lncRNA, the number of HCT116-specific SEs harbouring at least one of its correlated peaks. Of 4145 lncRNAs, 2136 had at least one overlapping SE. Among these, SNHG1—with 141 of 184 SEs—ranked in the 93rd percentile, but 162 lncRNAs showed comparable or greater overlap. The addition of the RNA–chromatin contact layer narrows these 141 SEs to 21 Ψ-SEs, illustrating that epigenetic correlations alone do not prioritise SNHG1, and that the intersection with physical contact data is required to delineate the Ψ-SE set (Figure 3B).

Consistent with this, Ψ-SEs harboured significantly more SNHG1 contact peaks than other contacted SEs (median: 2 vs. 1 peak per SE; Mann–Whitney U test, *p* = 0.011), indicating that the epigenetic association with SNHG1 at these loci is accompanied by a higher density of physical RNA–chromatin contacts.

The histone modification landscape of Ψ-SEs is dominated by positively correlated H3K4me1, present at 12 of 21 Ψ-SEs (57.1%) (Figure 2B). H3K4me1 is an enhancer-associated mark found at both active and primed/permissive enhancers [12], whereas H3K27ac is generally regarded as a more specific hallmark of fully active enhancers. The positive association of H3K4me1 with SNHG1 expression suggests that higher SNHG1 levels are associated with a more active enhancer chromatin state at these loci. The next most frequent marks were H3K4me3 with negative correlation (4 SEs, 19.0%) and H3K4me3 with positive correlation (3 SEs, 14.3%), followed by H3K4me1 (–) at 2 SEs and single instances of H3K27ac (+), H3K27me3 (+), and H3K36me3 (+). Overall, 18 of 24 mark instances (75%) showed positive correlations, indicating a predominant association of SNHG1 with active chromatin signatures. Three Ψ-SEs carried compound signatures: se_41 and se_12 displayed both H3K4me3 (+) and H3K4me1 (+), while se_75 carried H3K27ac (+) and H3K4me1 (+).

The near-absence of H3K27ac from the HiMoRNA-derived mark set at Ψ-SEs raises the question of whether these loci are in a fully active state. To address this directly, we intersected Ψ-SE coordinates with H3K27ac and H3K4me1 ChIP-seq data from ENCODE for HCT116 cells (ENCSR000EUT, ENCSR000EUS; replicated peaks, GRCh38). All 21 Ψ-SEs carried both H3K27ac and H3K4me1 peaks, with a mean H3K27ac density of 9.0 peaks per Ψ-SE compared to 7.6 per Ω-SE (Table S4), confirming that these super-enhancers are in a fully active chromatin state in HCT116 cells.

The low representation of H3K27ac in the HiMoRNA-derived mark set therefore reflects the cross-cell-type nature of the correlations rather than the absence of this modification. H3K27ac is characteristically high at super-enhancer loci across most cell types, resulting in low inter-tissue variance and consequently weak correlations with lncRNA expression. H3K4me1, by contrast, marks enhancer identity and exhibits greater variation across cell types, enabling detection of its correlation with SNHG1 expression. The Ψ-SE classification thus identifies super-enhancers at which enhancer identity—rather than momentary activity—covaries with SNHG1 levels across cellular contexts.

The co-occurrence of positive correlations with enhancer-type (H3K4me1) and promoter-type (H3K4me3) marks at se_41 and se_12 is notable. Super-enhancers that carry both marks have been associated with particularly robust transcriptional output [1,27,28], and the positive correlation of both with SNHG1 expression suggests that SNHG1 presence at these loci is associated with a dual-marked, highly active chromatin state.

The negative correlations with H3K4me3 at four Ψ-SEs (se_1, se_69, se_137, se_222) present a different scenario. At these loci, higher SNHG1 expression is associated with reduced H3K4me3, which may reflect a shift from a promoter-like to a more purely enhancer-like chromatin configuration. This is consistent with the reported ability of SNHG1 to interact with PRC2/EZH2 and modulate repressive histone marks [29], although the precise mechanism at these SE loci remains to be established experimentally.

Notably, 11 of the 21 Ψ-SEs showed concordant SNHG1-correlated histone modifications at both the SE body and the TSS of their target genes, as determined by parallel intersection of HiMoRNA peaks with target gene promoter regions. This SE–TSS concordance suggests coordinated epigenetic regulation spanning from enhancer to promoter at these loci.

Four Ψ-SEs (se_75, se_168, se_220, se_240) carried SNHG1-correlated H3K4me1 marks but lacked expressed target genes within the 100 kb proximity window. Inspection of these loci revealed distinct genomic contexts: se_168 (chr2) lies 53 kb from IRS1 (Insulin Receptor Substrate 1; TPM = 22.4 in HCT116); se_240 (chr8) lies 114 kb from TRIB1 (Tribbles Pseudokinase 1; TPM = 113.8) and 258 kb from SQLE (Squalene Epoxidase; TPM = 63.3); and se_220 (chr6) resides in a gene desert with no protein-coding gene within 450 kb. These cases suggest that a subset of Ψ-SEs may regulate distal targets through long-range chromatin interactions not captured by proximity-based gene assignment.

### 2.3. Target Genes of Ψ Super-Enhancers

Of the 21 Ψ-SEs, 17 had at least one expressed protein-coding target gene (TPM > 1) within 100 kb of the SE boundary, yielding 66 SE–gene associations (Table S5, Figure 2C,D). For each Ψ-SE, the lead gene was defined as the target with the lowest DESeq2 adjusted *p*-value in the comparison of HCT116 cells versus normal colon organoids (Table 1).

Differential expression analysis revealed a heterogeneous pattern among lead genes: 9 of 17 were significantly upregulated in HCT116 (DESeq2 padj < 0.05, log_2_FC > 0), 6 were significantly downregulated, and 2 did not reach significance. This bidirectionality indicates that Ψ-SEs are not uniformly associated with transcriptional activation, consistent with the presence of both positive and negative HiMoRNA correlations among the epigenetic marks at these loci. Two loci demonstrate this complexity: at se_13 (chr1, H3K4me3 (+)), the lead gene by statistical significance, C1orf210 (log_2_FC = −3.82), is strongly downregulated despite the positive SNHG1–H3K4me3 correlation at the SE body. However, two other genes within the same SE—CDC20 and EBNA1BP2—are upregulated and triple-validated (see below), illustrating that a single SE can harbour target genes with divergent transcriptional responses. At se_222 (chr6, H3K4me3 (−)), the lead gene SYTL3 is markedly upregulated (log_2_FC = +6.06) despite a negative SNHG1–H3K4me3 correlation. This apparent discordance may reflect the fact that the HiMoRNA mark captures a cross-tissue correlation with one specific histone modification, whereas gene expression in a given cell line integrates the net effect of multiple regulatory inputs. These cases underscore that the Ψ-SE classification identifies loci of SNHG1–chromatin co-occurrence, not a uniform direction of transcriptional effect.

To identify the most robust candidates, we applied a triple validation filter requiring concordant evidence across three independent data layers: upregulation in HCT116 (DESeq2 padj < 0.05, log_2_FC > 0), upregulation in TCGA-COAD tumors versus adjacent normal tissue (log_2_FC > 0, *p* < 0.05), and positive Spearman correlation with SNHG1 expression across 471 TCGA-COAD tumor samples. Twelve genes from seven Ψ-SEs passed all three criteria (Table 2).

The strongest case is presented by se_236 (chr8, H3K27me3 (+)), the only Ψ-SE at which all three target genes—*PDP1* (log_2_FC = +5.34), *TMEM67* (+1.99), and *RBM12B* (+1.59)—are triple-validated. PDP1 encodes Pyruvate Dehydrogenase Phosphatase 1, which activates the pyruvate dehydrogenase complex and controls pyruvate entry into the TCA cycle; its marked upregulation in HCT116 is notable given the role of PDP1 in mitochondrial pyruvate metabolism [30]. Notably, se_236 carries H3K27me3 with positive SNHG1 correlation—the only repressive mark in the Ψ-SE set with this direction—suggesting a distinct regulatory mechanism at this locus. At the same time, the positive correlation between SNHG1 expression and H3K27me3 at this locus coexists with high levels of active marks H3K4me1 and H3K27ac in HCT116 ChIP-seq data (Figure S1), indicating that the absence of H3K27ac from the SNHG1-correlated HiMoRNA assignment does not imply the absence of an active enhancer environment in HCT116. Together, these features suggest a bivalent or transitional chromatin configuration that has been described at cancer-associated regulatory elements [31] and may reflect incomplete resolution of Polycomb-mediated silencing during oncogenic reprogramming.

A second notable cluster is se_13 (chr1, H3K4me3 (+)), which illustrates functional diversity within a single Ψ-SE. The lead gene by statistical significance is C1orf210 (log_2_FC = −3.82, padj = 1.3 × 10^−36^), which is strongly downregulated. However, the same SE also harbours CDC20 (log_2_FC = +1.76) and EBNA1BP2 (+0.88), both triple-validated. CDC20 is a critical activator of the anaphase-promoting complex whose overexpression is a recognised feature of aggressive colorectal tumours, while EBNA1BP2 participates in ribosome biogenesis. The co-occurrence of strongly up- and downregulated genes within a single SE underscores that proximity-based assignment captures regulatory neighbours with potentially divergent transcriptional responses.

Among the remaining triple-validated genes, TOP1 (log_2_FC = +1.14, se_177, H3K4me1 (+)) is notable both as an essential DNA topoisomerase and as the molecular target of irinotecan, a first-line chemotherapeutic for colorectal cancer [32]. TXNRD1 (log_2_FC = +1.27, se_81, H3K4me1 (+)) contributes to redox homeostasis, and CWF19L1 and BLOC1S2 (se_41, H3K4me1 (+)/H3K4me3 (+)) are associated with a dual-marked SE carrying both enhancer- and promoter-type active modifications.

**Figure 4:**
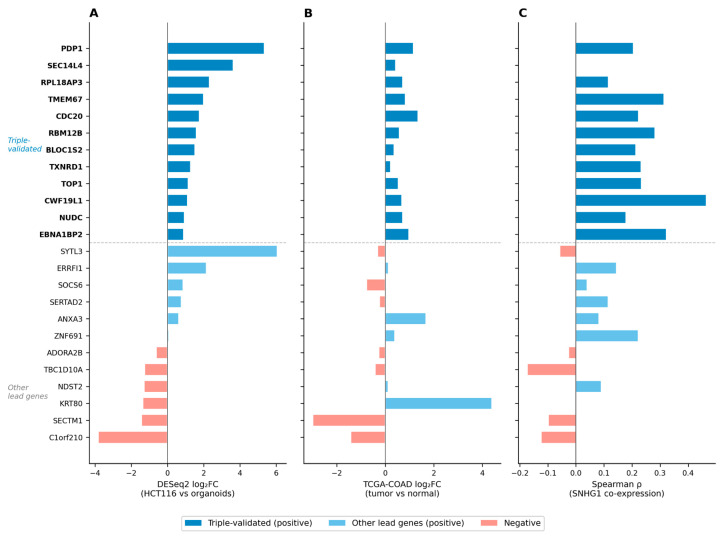
Multi-layer validation of Ψ-SE target genes. Horizontal bars show (**A**) DESeq2 log_2_FC (HCT116 vs. normal colon organoids), (**B**) TCGA-COAD log_2_FC (471 tumor vs. 41 adjacent normal), and (**C**) Spearman ρ between gene expression and SNHG1 across 471 TCGA-COAD tumors. The dashed line separates triple-validated genes (top) from other lead genes (bottom).

The 12 triple-validated genes span diverse functional categories (Table 2, Figure 4). Four have established roles in CRC biology: CDC20, an APC/C co-activator and independent prognostic factor in CRC [33]; TOP1, the molecular target of irinotecan and a candidate predictive biomarker for irinotecan response [32]; TXNRD1, a druggable redox enzyme whose inhibition by auranofin is cytotoxic to CRC cells [34]; and PDP1, recently identified as a scaffold for BRAF–MEK1 signaling in KRAS-mutant CRC [35]. The remaining genes include regulators of ribosome biogenesis (EBNA1BP2), mitotic spindle function (NUDC), endosomal trafficking (BLOC1S2), and RNA splicing (CWF19L1), while RBM12B and RPL18AP3 remain uncharacterized in cancer. Notably, these four CRC-relevant genes were identified solely through the epigenomic pipeline, without prior knowledge of their function, providing indirect support that the Ψ-SE framework captures biologically and clinically relevant targets. None of the 12 genes has been previously reported as a regulatory target of SNHG1 in any context, despite extensive literature on SNHG1 in CRC [29]. Their identification through the Ψ-SE framework nominates them as novel candidates for experimental validation.

### 2.4. TCGA-COAD Co-Expression Results

To assess whether the association between SNHG1 and Ψ-SE target genes extends beyond the HCT116 cell line, we examined SNHG1 expression and its co-expression with target genes in the TCGA-COAD cohort (471 tumor and 41 adjacent normal samples). SNHG1 was significantly upregulated in tumors (log_2_FC = 1.87, *p* = 5.9 × 10^−28^, Welch’s *t*-test on log_2_(TPM + 1) values), consistent with previous reports of SNHG1 overexpression in colorectal cancer [23]. Of the 66 Ψ-SE–gene associations, 65 genes were evaluable in the TCGA-COAD expression matrix (one gene lacked a matching HGNC identifier after Ensembl mapping).

We then computed Spearman correlations between SNHG1 and each of the 65 Ψ-SE target genes across the 471 tumor samples. Thirty of 65 evaluable genes (46.2%) showed significant positive correlations (*p* < 0.05; 29 at Benjamini–Hochberg FDR < 0.05), with a median ρ of 0.082 (Table S5). To determine whether this exceeds chance expectation, we performed a permutation test comparing the observed co-expression to 10,000 random gene sets of equal size drawn from the genome. Ψ-SE target genes showed significantly stronger co-expression with SNHG1 than random genes (median ρ = 0.082 vs. 0.035 expected, empirical *p* = 0.001; 30 significant positive correlations vs. 19.0 ± 3.7 expected, *p* = 0.003). To control for the potential confound of tumor purity, we repeated the analysis using partial Spearman correlations with ABSOLUTE-derived purity estimates for 444 matched samples. The results were consistent: median partial ρ = 0.074, permutation *p* = 0.014, confirming that the co-expression signal is not driven by differences in tumor cell content.

These results indicate that Ψ-SE target genes are co-expressed with SNHG1 in patient tumors more strongly than expected by chance. The TCGA data thus provide independent support for the cancer relevance of the identified target genes. Of note, a comparable co-expression signal is also observed for target genes of other SE classes, consistent with the general transcriptional coherence of SE-proximal genes in cancer. The specificity of the Ψ-SE classification therefore rests on the epigenomic evidence presented in Section 2.1 and Section 2.2, rather than on co-expression alone.

Notably, the Ψ/Ω classification defined in Section 2.2 is based on the presence of SNHG1-confirmed HiMoRNA peaks within the SE body. A parallel analysis of target gene promoter regions revealed an additional layer of complexity: 30 Ω-SEs lacked SNHG1-correlated histone modifications on the SE body but harboured such marks at the TSS of one or more of their target genes. These cases suggest that the regulatory relationship between SNHG1 and certain SEs may be mediated through the promoter rather than the enhancer element itself.

The most informative of these loci is se_244, an Ω-SE on chr8 that maps to the 8q24 region harbouring the PVT1 lncRNA. PVT1 (TPM = 181.9 in HCT116) carries an H3K4me1 (+) SNHG1-correlated mark at its TSS, while the SE body shows no SNHG1-confirmed HiMoRNA peaks. PVT1 stabilises MYC protein and exhibits independent oncogenic activity; the presence of an SNHG1-correlated active mark at its promoter—but not at the governing SE—raises the possibility that SNHG1 influences PVT1 expression through promoter-proximal rather than enhancer-mediated mechanisms. Other notable cases include se_207, whose target genes AP3S1 and ATG12 carry H3K4me3 (+) marks at their TSS, and se_176, where EIF6 shows both H3K4me3 (+) and H3K27me3 (+) marks, indicative of a bivalent chromatin state.

The 21 Ψ-SEs and the 30 Ω-SE–TSS cases thus define complementary modes of SNHG1–super-enhancer association: one in which SNHG1-correlated epigenetic marks reside on the SE body, and another in which they reside at the target gene promoter. Whether these modes reflect distinct regulatory mechanisms or different stages of a dynamic process remains an open question.

### 2.5. Interpretation, Limitations, and Outlook

Several limitations of the current analysis should be acknowledged. First, the co-occurrence of SNHG1 contacts and SNHG1-correlated histone modifications at Ψ-SEs is consistent with a functional role for SNHG1 in shaping the SE chromatin environment, but does not demonstrate causality. The HiMoRNA correlations are derived from cross-cell-type comparisons, and the RNA-Chrom contact data represent population-averaged measurements. Because HiMoRNA correlations are computed across heterogeneous cell types and tissues, they may capture shared tissue-specificity patterns rather than direct functional relationships within a single cell line. Consequently, a positive SNHG1–histone mark correlation at a given locus does not imply that SNHG1 directly recruits or maintains that modification in HCT116 cells. Establishing whether SNHG1 directly modulates histone modifications at Ψ-SEs requires experimental validation through SNHG1 knockdown coupled with ChIP-seq or CUT&Tag in HCT116 cells [36].

Second, the analysis is based on a single cancer cell line, and the generalisability of the Ψ-SE set to other colorectal cancer models or primary tumours remains to be tested. Additionally, the normal colon reference used for differential expression analysis comprises organoid lines derived from a single pediatric donor, limiting biological generalisability. Furthermore, because these organoids were cultured under Wnt pathway stimulation, the baseline expression of Wnt-responsive genes may be elevated relative to native colon epithelium, potentially leading to underestimation of tumor-versus-normal fold changes for this gene set. Third, the proximity-based target gene assignment (100 kb window) may miss distal regulatory interactions; indeed, four Ψ-SEs lacked target genes within this window, suggesting long-range regulation that could be captured by chromatin conformation data. In particular, integration with publicly available Hi-C data for HCT116 [37] would enable validation of predicted SE–target gene interactions and resolution of these cases. Similarly, the tissue-specificity classification employed a default overlap threshold (≥1 bp); a more stringent reciprocal overlap criterion could reclassify a small number of SEs between categories, although the downstream Ψ-SE identification depends on two additional filtering layers and is therefore robust to minor boundary shifts.

Despite these caveats, the integrative framework offers several directions for further investigation. Among the target genes identified, TOP1 (the target of irinotecan) and PDP1 (a key metabolic enzyme) represent candidates where the connection between SE-level regulation and drug targets warrants further investigation. The pipeline itself is generalisable and can be applied to any lncRNA represented in both RNA-Chrom and HiMoRNA to systematically map lncRNA–SE regulatory axes across cell types and disease contexts. As RNA–chromatin interaction datasets expand, this approach may enable the construction of comprehensive lncRNA–SE regulatory maps for multiple cancer types.

## 3. Materials and Methods

### 3.1. Data Sources

#### 3.1.1. RNA–Chromatin Contact Data

RNA–chromatin contacts of SNHG1 in HCT116 colorectal cancer cells were obtained from the RNA-Chrom database [21]. The RNA-Chrom database provides genome-wide one-to-all experimental data mapping the interactions of specific RNA molecules with chromatin. For this study, we used Experiment ID: 130, which contains SNHG1 RNA–chromatin interaction data from HCT116 cells cultured under native conditions without additional experimental treatments. The underlying ChIRP-seq data were originally reported by Sun et al. [38] (GEO: GSE85842) and reprocessed within the RNA-Chrom pipeline. The experiment comprises three biological replicates, each sequenced using a split-probe design in which contacts are assigned to “odd” and “even” fractions based on alternating probe subsets; this design provides an internal reproducibility control for peak calling. In addition, matched input control libraries were available for each biological replicate and were used during MACS2 peak calling.

#### 3.1.2. Histone Modification Correlation Data

Data on correlations between lncRNA expression and histone modification levels were obtained from the HiMoRNA database [22]. HiMoRNA catalogues genomic loci where the intensity of histone modification ChIP-seq signal significantly correlates with lncRNA expression across multiple cell types and tissues. Statistical significance is assessed within the database by computing Spearman correlations with correction for multiple testing; only peaks passing the significance threshold are retained. For SNHG1, the database contains 90,586 peaks with significant correlations across seven histone marks (H3K4me1, H3K4me3, H3K27ac, H3K27me3, H3K36me3, H3K9me3, H4K20me1), each annotated with the direction of correlation (positive or negative). The full database dump (himorna_database_dump_peaks_and_correlations.tar.gz) and the lncRNA correspondence table (lncRNA_CorrespondenceTable.csv) were downloaded for analysis. For the lncRNA comparison analysis (Section 2.2), HiMoRNA peaks for all 4145 catalogued lncRNAs were used. To assess the chromatin state of Ψ-SEs independently of HiMoRNA, H3K27ac and H3K4me1 ChIP-seq peak sets for HCT116 cells were obtained from ENCODE (H3K27ac: ENCSR000EUT, file ENCFF328BFB; H3K4me1: ENCSR000EUS, file ENCFF657QSG; replicated peaks, GRCh38).

#### 3.1.3. Super-Enhancer Annotations

Three HCT116 H3K27ac ChIP-seq samples (SE_01_0034, SE_02_1029, SE_02_0352) and six normal colon tissue samples (SE_00_0035, SE_01_0083, SE_01_0089, SE_01_0099, SE_01_0109, SE_02_0477) were retrieved from SEdb 3.0 [11]; original H3K27ac ChIP-seq source accessions for each sample are documented within the database. All SE coordinates were provided in the hg38 (GRCh38) genome assembly.

#### 3.1.4. Transcriptomic Data

For HCT116 gene expression quantification, preprocessed RNA-seq data counts were obtained from the Gene Expression Omnibus (accessions GSM5904712 and GSM5904713), representing two biological replicates. Gene expression was quantified as transcripts per million (TPM). For normal colon tissue, preprocessed RNA-seq data counts were obtained from the ENCODE project [39] (accessions ENCFF367ZVO, ENCFF366BBV, ENCFF801VQX, ENCFF620DRS, and ENCFF490WXZ), comprising five samples. Expression levels were quantified as TPM. The mean TPM across available replicates was used for both HCT116 and normal tissue for target gene identification.

#### 3.1.5. TCGA-COAD Data

Gene expression data for colon adenocarcinoma (TCGA-COAD) were downloaded from the UCSC Xena platform [40] as log_2_(TPM + 1) values (dataset: TCGA-COAD.star_tpm.tsv.gz; 471 primary tumor and 41 solid tissue normal samples). Gene identifiers were mapped from Ensembl to HGNC symbols using the gencode.v36.annotation.gtf.gene.probemap file. Consensus tumor purity estimates were obtained from Aran et al. [41] (ABSOLUTE algorithm), providing purity values for 444 of the 471 tumor samples.

### 3.2. Construction of the Consensus Super-Enhancer Set

A consensus super-enhancer set was constructed independently for HCT116 and normal colon tissue. For each condition, SE coordinates from the respective SEdb 3.0 datasets (Section 3.1.3) were first consolidated within each individual sample by merging adjacent SEs separated by less than 12,500 bp using bedtools merge [42]. A consensus annotation was then generated by applying bedtools merge across all samples for a given condition, retaining only merged regions supported by all three samples for HCT116 and by at least five of six samples for a normal colon. This procedure yielded 571 consensus super-enhancers in total. By comparing the HCT116 and normal colon consensus sets using bedtools merge with default parameters (minimum overlap ≥ 1 bp), SEs were classified as HCT116-specific (n = 184), normal colon-specific (n = 247), or shared (n = 140). Genomic coordinates and classifications for all 571 consensus SEs are provided in Table S3.

### 3.3. SNHG1 RNA–Chromatin Contact Peak Calling

SNHG1 RNA–chromatin contacts from the RNA-Chrom database (Exp. ID: 130) were processed using MACS2 v2.2.6 [43] and BEDTools v2.30.0 [42]. Contact intervals from the odd, even, and input fractions of each biological replicate were first sorted and filtered against the ENCODE hg38 blacklist (ENCFF356LFX) using bedtools intersect -v. Peak calling was then performed separately for the odd and even fractions of each biological replicate using the corresponding replicate-matched input fraction as control, with MACS2 parameters -f BEDPE -g hs --keep-dup all; all other parameters were left at default values. This procedure yielded one odd and one even peak set for each of the three biological replicates.

To derive replicate-level supported contact peaks, the odd and even peak sets from the same biological replicate were compared as follows. First, odd and even narrowPeak intervals were symmetrically extended by 250 bp on each side using bedtools slop. Second, the expanded odd and even intervals were intersected using bedtools intersect. For each intersecting odd/even peak pair, a replicate-supported interval was reconstructed from the original coordinates as start = min(start_even, start_odd) + 250 and end = max(end_even, end_odd) − 250. Thus, only peak pairs with direct overlap or separated by no more than 500 bp were retained. The resulting replicate-supported intervals were then merged within each replicate using bedtools merge.

To obtain the final consensus contact set, replicate-level peak sets were compared pairwise across the three biological replicates using bedtools intersect. Peaks supported by at least two of the three biological replicates were retained, pooled, sorted, and merged with bedtools merge to generate the final consensus peak set, yielding 17,027 high-confidence SNHG1 contact peaks (Table S6).

### 3.4. Data Integration Pipeline

The integration of SNHG1 contact data, HiMoRNA correlation data, and super-enhancer annotations proceeded in two stages. In the first stage, the 90,586 HiMoRNA peaks for SNHG1 were intersected with the 17,027 SNHG1 MACS2 contact peaks using bedtools intersect with the “-u” parameter, yielding 2640 SNHG1-confirmed HiMoRNA peaks—genomic loci where SNHG1 physical contact and SNHG1-correlated histone modification co-occur (Table S7). In the second stage, the 2640 SNHG1-confirmed HiMoRNA peaks were intersected with the 184 HCT116-specific super-enhancers using bedtools intersect. SEs containing at least one SNHG1-confirmed HiMoRNA peak were classified as Ψ-SE (SNHG1-reactive); the remainder were classified as Ω-SE (SNHG1-independent).

### 3.5. Target Gene Identification

For each consensus super-enhancer, target genes were identified using two criteria: (1) the gene must be expressed in HCT116 cells (mean TPM > 1 across replicates); and (2) the start coordinate of the gene must be located within 100 kb of the SE boundary, or the gene must overlap the SE. Gene annotations were based on GENCODE v36 [44]. This approach yielded 66 Ψ-SE–gene associations across the 17 Ψ-SEs with expressed target genes.

### 3.6. Differential Expression Analysis

Differential expression between HCT116 and normal colon epithelium was assessed using DESeq2 (v1.34) [45] on gene-level counts. HCT116 count data were obtained from GSE196921 (control condition, pSin empty vector; n = 2 biological replicates) [46]; normal colon count data were obtained from GSE266990, comprising four wild-type intestinal organoid lines derived from sigmoid colon biopsies of a single pediatric donor. Mitochondrial genes, Y-chromosome genes, and the XIST transcript were computationally excluded from the raw count matrices prior to analysis. Pre-filtering was applied to retain only genes with at least 10 counts in a minimum of two samples, corresponding to the smallest experimental group size (n = 2). Normalization via size factor estimation and subsequent differential expression analysis were performed using the DESeq2 package with default parameters, and *p*-values were adjusted for multiple testing using the Benjamini–Hochberg procedure. Genes with padj < 0.05 were considered differentially expressed. To maximize statistical power and reduce the false discovery rate (FDR) penalty during independent filtering, hypothesis testing was strictly restricted to genes annotated with protein-coding or lncRNA biotypes.

For independent validation in patient samples, differential expression between tumor and adjacent normal tissue in the TCGA-COAD cohort was assessed using Welch’s *t*-test on log_2_(TPM + 1) values (471 tumor vs. 41 normal samples). This analysis was performed genome-wide (56,534 genes) to enable comparison across all SE target gene classes.

### 3.7. TCGA-COAD Co-Expression Analysis

To assess whether Ψ-SE target genes are co-expressed with SNHG1 in patient tumors, Spearman rank correlations were computed between SNHG1 and each target gene across 471 TCGA-COAD primary tumor samples. *p*-values were adjusted using the Benjamini–Hochberg procedure. To evaluate whether the observed co-expression exceeds chance expectation, 10,000 random gene sets of equal size (n = 65) were drawn from all expressed genes in the TCGA-COAD matrix, and the same metrics (number of significant positive correlations, median ρ, mean ρ) were computed for each random set. Empirical *p*-values were calculated as the fraction of permutations with values equal to or greater than the observed. To control for tumor purity as a potential confound, partial Spearman correlations were computed. For each gene, SNHG1 expression and gene expression were rank-transformed, linearly regressed against rank-transformed ABSOLUTE purity estimates, and the Pearson correlation of the residuals was used as the partial correlation coefficient. This analysis was restricted to 444 tumor samples with available purity data. The permutation test was repeated on purity-corrected correlations.

### 3.8. HiMoRNA lncRNA Comparison

To contextualise SNHG1 among other lncRNAs, HiMoRNA peaks for all 4145 lncRNAs in the database were intersected with the 184 HCT116-specific SE coordinates using coordinate overlap. For each lncRNA, the number of SEs harbouring at least one HiMoRNA peak was counted. The resulting distribution was used to determine the percentile rank of SNHG1.

### 3.9. Statistical Analysis

To assess the significance of the SNHG1–HiMoRNA–SE triple intersection, a joint permutation test was performed. In each of 1000 iterations, the 17,027 SNHG1 MACS2 peaks were shuffled across the genome using bedtools shuffle (-chrom -noOverlapping), preserving peak sizes and chromosomal assignment. The full set of 90,586 SNHG1-correlated HiMoRNA peaks was then intersected with the shuffled contact peaks (bedtools intersect -a himorna_peaks.bed -b shuffled_contacts.bed -u) to obtain the iteration-specific set of “confirmed” HiMoRNA peaks. These were subsequently intersected with the 184 HCT116-specific super-enhancers to count the number of SEs carrying at least one confirmed peak. The empirical *p*-value was calculated as the fraction of permutations yielding a count equal to or greater than the observed value. To assess enrichment of SNHG1 contacts at SE loci, a separate permutation test was performed using the same shuffling procedure, counting the number of HCT116-specific SEs overlapping at least one shuffled peak per iteration. To compare SNHG1 contact density between Ψ-SE and other contacted super-enhancers, the number of overlapping SNHG1 peaks per SE was computed using bedtools intersect and compared by a one-sided Mann–Whitney U test. *p*-values < 0.05 were considered significant throughout. The reference genome assembly hg38 (GRCh38) was used throughout.

## 4. Conclusions

By integrating RNA–chromatin contact data, histone modification correlation profiles, and super-enhancer annotations across three public databases, we identified 21 SNHG1-reactive super-enhancers (Ψ-SEs) among 184 HCT116-specific Ses. These are a defined subset at which SNHG1 physical contacts co-occur with SNHG1-correlated epigenetic modifications, predominantly H3K4me1, which is an enhancer-associated mark reflecting active as well as permissive enhancer states. Comparison with 4145 lncRNAs in the HiMoRNA database demonstrated that neither epigenetic correlations nor chromatin contacts alone are sufficient to delineate this subset, underscoring the value of the triple-intersection approach.

Differential expression analysis identified 12 target genes across seven Ψ-SEs that are consistently upregulated both in HCT116 cells and in TCGA-COAD patient tumors and positively correlated with SNHG1 expression, including CDC20, PDP1, TOP1, and TXNRD1. Co-expression analysis in 471 TCGA-COAD tumors confirmed that Ψ-SE target genes are more strongly associated with SNHG1 than expected by chance, a signal robust to correction for tumor purity.

The Ψ/Ω classification introduced here provides a framework for distinguishing super-enhancers at which lncRNA–chromatin interactions may contribute to the local epigenetic environment from those that operate independently. While these findings are correlative and require experimental validation through SNHG1 perturbation coupled with epigenomic profiling, the integrative pipeline is readily applicable to other lncRNAs catalogued in RNA-Chrom and HiMoRNA, offering a scalable approach for systematic mapping of lncRNA–super-enhancer regulatory axes in cancer and beyond.

## Figures and Tables

**Table 1:** Lead target genes of SNHG1-reactive super-enhancers (Ψ-SEs). For each Ψ-SE with expressed protein-coding target genes, the gene with the lowest DESeq2 adjusted *p*-value (HCT116 vs. normal colon tissue) is shown. log_2_FC and padj are from DESeq2 analysis.

Ψ-SE	Chr	HiMoRNA Mark	Lead Gene	log_2_FC	padj
se_1	chr1	H3K4me3 (−)	ERRFI1	2.14	5.6 × 10^−27^
se_6	chr1	H3K4me1 (+)	NUDC	0.93	4.3 × 10^−6^
se_12	chr1	H3K4me1 (+), H3K4me3 (+)	ZNF691 *	0.07	8.7 × 10^−1^
se_13	chr1	H3K4me3 (+)	C1orf210	−3.82	1.3 × 10^−36^
se_35	chr10	H3K4me1 (+)	NDST2	−1.28	5.5 × 10^−10^
se_41	chr10	H3K4me1 (+), H3K4me3 (+)	CWF19L1	1.10	5.9 × 10^−7^
se_69	chr12	H3K4me3 (−)	KRT80	−1.36	7.5 × 10^−5^
se_81	chr12	H3K4me1 (+)	TXNRD1	1.27	8.2 × 10^−8^
se_110	chr17	H3K4me1 (+)	ADORA2B	−0.62	8.8 × 10^−4^
se_137	chr17	H3K4me3 (−)	SECTM1	−1.43	1.1 × 10^−5^
se_140	chr18	H3K36me3 (+)	SOCS6	0.86	1.5 × 10^−4^
se_157	chr2	H3K4me1 (+)	SERTAD2	0.77	3.2 × 10^−4^
se_177	chr20	H3K4me1 (+)	TOP1	1.14	3.2 × 10^−22^
se_184	chr22	H3K4me1 (+)	TBC1D10A	−1.26	3.3 × 10^−9^
se_202	chr4	H3K4me1 (+)	ANXA3 *	0.62	8.3 × 10^−2^
se_222	chr6	H3K4me3 (−)	SYTL3	6.06	2.3 × 10^−75^
se_236	chr8	H3K27me3 (+)	PDP1	5.34	4.9 × 10^−144^
se_75	chr12	H3K27ac (+), H3K4me1 (+)	— ^†^	—	—
se_168	chr2	H3K4me1 (+)	— ^†^	—	—
se_220	chr6	H3K4me1 (+)	— ^†^	—	—
se_240	chr8	H3K4me1 (+)	— ^†^	—	—

* se_12: ZNF691 (padj = 0.87) and se_202: ANXA3 (padj = 0.083) did not reach statistical significance (padj < 0.05); included as the lowest-padj genes within their respective Ψ-SE windows. ^†^ No expressed protein-coding gene (TPM > 1) within 100 kb. Nearest genes: se_168—*IRS1* (53 kb); se_240—*TRIB1* (114 kb); se_220—gene desert (>450 kb); se_75—no proximal target.

**Table 2:** Triple-validated Ψ-SE target genes: TCGA-COAD co-expression and functional annotation.

Gene	Ψ-SE	TCGA log_2_FC	SNHG1 ρ	FDR	Molecular Function	Cancer Relevance
PDP1	se_236	1.153	0.2044	<0.001	Pyruvate dehydrogenase phosphatase; activates PDH complex	Scaffold for BRAF–MEK1 in KRAS-mutant CRC ^1^; prognostic marker
CDC20	se_13	1.349	0.2226	<0.001	APC/C co-activator; essential for anaphase onset	Overexpressed in CRC; independent prognostic factor; radiosensitization target
TOP1	se_177	0.531	0.2331	<0.001	DNA topoisomerase I; relaxes supercoiled DNA	Molecular target of irinotecan (first-line mCRC); chr20q amplification biomarker
TXNRD1	se_81	0.219	0.2322	<0.001	Thioredoxin reductase 1; cytosolic redox homeostasis	NRF2 target; druggable (auranofin); unfavorable prognosis pan-cancer
TMEM67	se_236	0.82	0.3131	<0.001	Ciliary transition zone receptor; Wnt signaling modulator	No direct CRC data; regulates canonical Wnt pathway
EBNA1BP2	se_13	0.964	0.3223	<0.001	Nucleolar scaffold; 60S ribosome subunit maturation	c-Myc positive feedback loop; p53 destabilization
CWF19L1	se_41	0.674	0.4646	<0.001	Spliceosome-associated; RNA lariat debranching	Enhances CD8+ T cell cytotoxicity against colon carcinoma (MC38)
NUDC	se_6	0.715	0.1784	<0.001	Dynein complex regulator; mitotic spindle and cytokinesis	Family member NudCD1 is prognostic in CRC; upregulated in leukemia
BLOC1S2	se_41	0.357	0.2129	<0.001	BLOC-1/BORC subunit; endosomal–lysosomal trafficking	Negatively regulates Notch1 via lysosomal degradation
RBM12B	se_236	0.581	0.2814	<0.001	RNA-binding motif protein 12B; putative mRNA processing	Uncharacterized in cancer
SEC14L4	se_184	0.428	0.0023	0.9600	SEC14-family lipid-binding protein	Oncogenic in ESCC ^1^ via MAPK activation; no CRC data
RPL18AP3	se_81	0.716	0.1154	0.0223	Processed pseudogene of RPL18A; potential ceRNA	Uncharacterized in cancer

^1^ CRC, colorectal cancer; ESCC, esophageal squamous cell carcinoma.

## Data Availability

All primary data analyzed in this study are publicly available from the following sources: RNA-Chrom (https://rnachrom2.bioinf.fbb.msu.ru/, accessed on 14 April 2026), HiMoRNA (https://himorna.fbras.ru/, accessed on 14 April 2026), SEdb 3.0 (http://www.licpathway.net/sedb/, accessed on 14 April 2026), and TCGA-COAD (https://portal.gdc.cancer.gov/, accessed on 14 April 2026). Data generated during the analysis, including the final consensus SNHG1 contact peak set, the SNHG1 HiMoRNA peak list, the SNHG1-confirmed HiMoRNA overlap set, SE classifications, and co-expression matrices, are provided in the Supplementary Materials. The analysis code and pipeline are available at https://github.com/comp-biomed-lab/SNHG1, accessed on 14 April 2026.

## Supplementary Materials

The following supporting information can be downloaded at: https://www.mdpi.com/article/10.3390/ijms27083642/s1.
