# Supplementary material for: Integrative Mapping of SNHG1 RNA–Chromatin Contacts onto the Cancer-Specific Super-Enhancer Landscape in HCT116 Colorectal Cancer Cells
Source: Int J Mol Sci. 2026 Apr 19;27(8):3642. doi: 10.3390/ijms27083642 (PMC13115861; doi:10.3390/ijms27083642)
Supplement: Supplementary file 1 [file ijms-27-03642-s001.zip › Supplementary Materials.pdf]

Supplementary Materials

Integrative mapping of SNHG1 RNA–chromatin contacts onto the cancer-specific super-enhancer landscape in HCT116 colorectal cancer cells

Grigory K. Ryabykh [1,†], Ekaterina D. Osintseva [2,†], German A. Ashniev [2,3], Yulia V. Makus [2,3], Alexey V. Orlov [2,3*], Petr I. Nikitin [2], and Natalia N. Orlova [2,*]

1 Vavilov Institute of General Genetics, Russian Academy of Sciences, 3 Gubkina St., 119991 Moscow, Russia;
2 Prokhorov General Physics Institute of the Russian Academy of Sciences, 38 Vavilov St., 119991 Moscow, Russia;
3 Central University, 7 Gasheka St., 125212 Moscow, Russia
* Correspondence: alexey.orlov@kapella.gpi.ru (A.V.O.); natalja191186@gmail.com (N.N.O.)
† These authors contributed equally to this work.

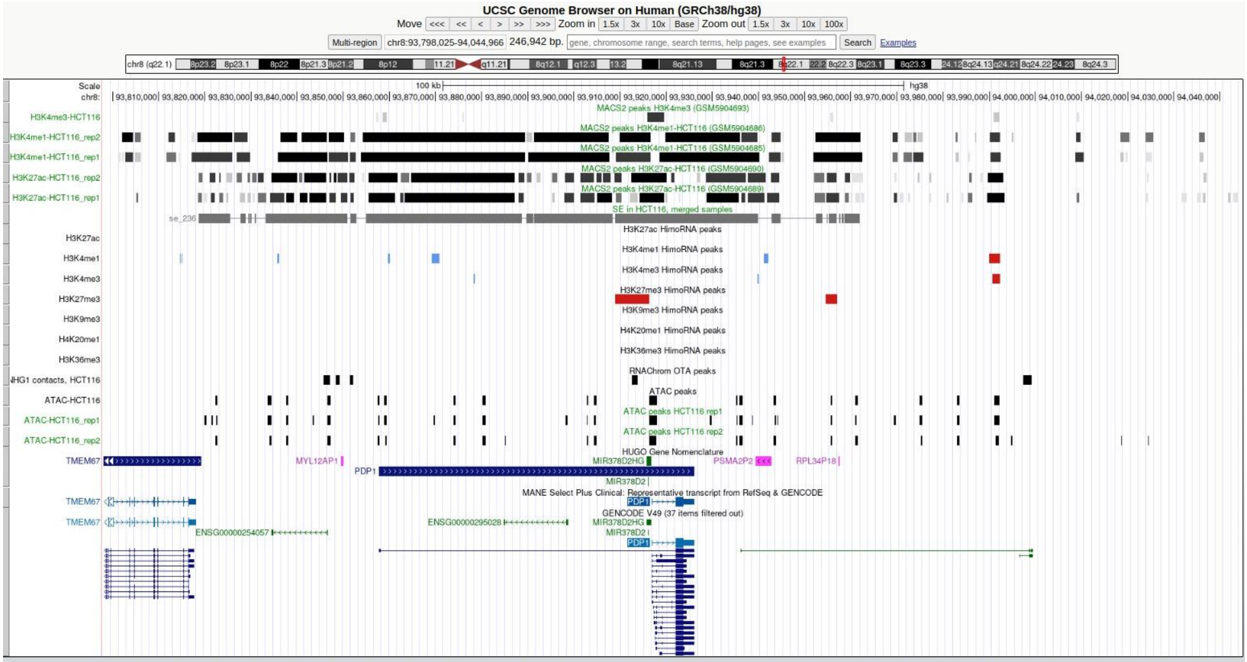

Figure S1. Chromatin landscape at the se_236 locus (chr8:93,798,025–94,044,966; hg38). UCSC Genome Browser view showing: (top) ChIP-seq signal tracks for H3K4me3, H3K4me1, and H3K27ac in HCT116 cells with corresponding MACS2 peak calls; (middle) the se_236 super-enhancer annotation from SEdb 3.0 (merged HCT116 samples), followed by SNHG1-correlated HiMoRNA peaks for seven histone marks — notably, the H3K27me3 HiMoRNA peak (red) overlaps with the SNHG1 RNA–chromatin contact region (RNAChrom OTA peaks); (bottom) ATAC-seq open chromatin peaks in HCT116 and GENCODE gene annotations, including PDP1, TMEM67, and RBM12B. The co-occurrence of the H3K27me3 HiMoRNA peak with active ChIP-seq marks (H3K4me1, H3K27ac) at this locus is consistent with a bivalent or transitional chromatin configuration.

Table S1. Full list of SNHG1 HiMoRNA peaks used in the analysis. For SNHG1, the HiMoRNA database contains 90,586 genomic loci where histone modification ChIP-seq signal significantly correlates with SNHG1 expression across cell types and tissues. Columns include: chromosome, start, end, histone mark, correlation coefficient, and peak identifier.

Provided as a separate Excel file (Table_S1.xlsx).

Table S2. SNHG1 contact enrichment at enhancer classes. Permutation test results (1,000 iterations, bedtools shuffle) comparing observed SNHG1 contact overlap with expected overlap for four enhancer categories: HCT116-specific super-enhancers, H3K27ac-defined typical enhancers, H3K4me1-defined typical enhancers, and all typical enhancers (union). H3K27ac and H3K4me1 ChIP-seq peaks for HCT116 were obtained from ENCODE (ENCSR000EUT, ENCSR000EUS; replicated peaks, GRCh38); typical enhancers were defined by excluding super-enhancer regions. Columns include: category, number of regions, mean region size, total genomic coverage, observed overlap count, percentage overlap, expected overlap (mean ± SD), fold enrichment, and empirical p-value.

Provided as a separate file (Table_S2.csv).

Table S3. HCT116-specific consensus super-enhancer coordinates and Ψ/Ω classification. Genomic coordinates and classification of 184 HCT116-specific consensus super-enhancers derived from the integration of three SEdb 3.0 H3K27ac ChIP-seq annotations. Columns include: genomic coordinates (hg38), SE identifier, source tissue/cell line, and Ψ/Ω classification. SE boundaries were defined by merging adjacent annotations within 12,500 bp and retaining regions supported by all three constituent datasets (see Section 3.2 of main manuscript).

Provided as a separate Excel file (Table_S3.xlsx).

Table S4. H3K27ac and H3K4me1 ChIP-seq peak overlap at Ψ-SE and Ω-SE classes. Direct assessment of the chromatin activation state at Ψ-SEs and Ω-SEs using ENCODE ChIP-seq data for HCT116 (H3K27ac: ENCSR000EUT, file ENCFF328BFB; H3K4me1: ENCSR000EUS, file ENCFF657QSG; replicated peaks, GRCh38). Columns include: SE class (Ψ-SE or Ω-SE), number of SEs, number overlapping H3K27ac peaks, number overlapping H3K4me1 peaks, and mean H3K27ac peak density per SE.

Provided as a separate file (Table_S4.csv).

Table S5. Complete list of Ψ-SE target genes with multi-layer validation data. For each of the 17 Ψ-SEs with expressed protein-coding target genes (TPM > 1 in HCT116), all associated genes within the 100 kb proximity window are listed (66 SE–gene associations in total). Columns include: Ψ-SE identifier, chromosome, HiMoRNA mark and direction of correlation, gene symbol, HCT116 expression (TPM), normal colon expression (TPM), DESeq2 $\log_2$FC and adjusted p-value (HCT116 vs normal colon organoids), TCGA-COAD $\log_2$FC (471 tumor vs 41 adjacent normal), TCGA-COAD differential expression p-value, Spearman ϱ between gene expression and SNHG1 across 471 TCGA-COAD tumor samples, Spearman correlation p-value, and triple-validation status (✓ indicates

genes passing all three criteria: DESeq2 upregulation, TCGA-COAD upregulation, and positive SNHG1 co-expression).

Provided as a separate Excel file (Table_S5.xlsx).

Table S6. Final consensus SNHG1 RNA–chromatin contact peaks. The 17,027 high-confidence SNHG1 contact peaks identified by MACS2 peak calling and reproducibility filtering across three biological replicates (see Section 3.3 of main manuscript). Columns include: chromosome, start, and end coordinates (hg38).

Provided as a separate Excel file (Table_S6.xlsx).

Table S7. SNHG1-confirmed HiMoRNA peaks overlapping the consensus SNHG1 contact set. The 2,640 HiMoRNA peaks that directly overlap with SNHG1 RNA–chromatin contact peaks, representing genomic loci where SNHG1 physical contact and SNHG1-correlated histone modification co-occur (see Section 3.4 of main manuscript). Columns include: chromosome, start, end, histone mark, correlation coefficient, and peak identifier.

Provided as a separate Excel file (Table_S7.xlsx).
